# An analysis of 2‐day cardiopulmonary exercise testing to assess unexplained fatigue

**DOI:** 10.14814/phy2.14564

**Published:** 2020-09-05

**Authors:** Jacob B. Lindheimer, Thomas Alexander, Wei Qian, Jacquelyn C. Klein‐Adams, Gudrun Lange, Benjamin H. Natelson, Dane B. Cook, Helene Z. Hill, Michael J. Falvo

**Affiliations:** ^1^ William S. Middleton Memorial Veterans Hospital Madison WI USA; ^2^ Department of Kinesiology University of Wisconsin‐Madison Madison WI USA; ^3^ Department of Medicine University of Wisconsin‐Madison Madison WI USA; ^4^ VA Airborne Hazards and Burn Pits Center of Excellence War Related Illness and Injury Study Center VA New Jersey Health Care System East Orange NJ USA; ^5^ New Jersey Medical School Rutgers Biomedical and Health Sciences Newark NJ USA; ^6^ Department of Neurology Icahn School of Medicine at Mount Sinai New York NY USA

**Keywords:** cardiopulmonary, exercise, exercise test, fatigue, fatigue syndrome, chronic, persian gulf fyndrome

## Abstract

Two consecutive maximal cardiopulmonary exercise tests (CPETs) performed 24 hr apart (2‐day CPET protocol) are increasingly used to evaluate post‐exertional malaise (PEM) and related disability among individuals with myalgic encephalomyelitis/chronic fatigue syndrome (ME/CFS). This protocol may extend to other fatiguing illnesses with similar characteristics to ME/CFS; however, 2‐day CPET protocol reliability and minimum change required to be considered clinically meaningful (i.e., exceeding the standard error of the measure) are not well characterized. To address this gap, we evaluated the 2‐day CPET protocol in Gulf War Illness (GWI) by quantifying repeatability of seven CPET parameters, establishing their thresholds of clinically significant change, and determining whether changes differed between veterans with GWI and controls. Excluding those not attaining peak effort criteria (*n* = 15), we calculated intraclass correlation coefficients (ICCs), the smallest real difference (SRD%), and repeated measures analysis of variance (RM‐ANOVA) at the ventilatory anaerobic threshold (VAT) and peak exercise in 15 veterans with GWI and eight controls. ICC values at peak ranged from moderate to excellent for veterans with GWI (mean [range]; 0.84 [0.65 – 0.92]) and were reduced at the VAT (0.68 [0.37 – 0.78]). Across CPET variables, the SRD% at peak exercise for veterans with GWI (18.8 [8.8 – 28.8]) was generally lower than at the VAT (28.1 [9.5 – 34.8]). RM‐ANOVAs did not detect any significant group‐by‐time interactions (all *p* > .05). The methods and findings reported here provide a framework for evaluating 2‐day CPET reliability, and reinforce the importance of carefully considering measurement error in the population of interest when interpreting findings.

## Graphical abstract

Two consecutive maximal cardiopulmonary exercise tests (CPETs) performed 24 hr apart (referred to as 2‐day CPET protocol) are increasingly promoted as an important clinical tool for illness diagnosis and measurement of disability in conditions with idiopathic fatigue; however, reliability and minimum values necessary to be considered clinically meaningful are not well characterized. With an emphasis on taking standard error of the measure into account, this study introduces a framework for evaluating repeatability and establishing reference values in the 2‐day CPET.

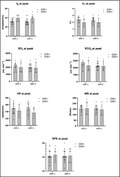

## 1 INTRODUCTION

Post‐exertional malaise (PEM) is defined as a worsening of symptoms following physical or mental activity and is a hallmark symptom of myalgic encephalomyelitis/chronic fatigue syndrome (ME/CFS) (Clayton, 2015). In addition to symptom exacerbation (e.g., fatigue, pain, and sleep disturbance), PEM is conceptualized as a notable decrement in functional capacity beyond what is expected in a healthy individual (Clayton, 2015). As a possible objective way to identify PEM, several investigators have promoted using a repeated‐bout cardiopulmonary exercise test (CPET) protocol whereby two maximal effort CPETs are performed 24 hr apart, referred to as a 2‐day CPET protocol (Stevens, Snell, Stevens, Keller, & VanNess, 2018). Findings from these studies may generally be summarized as follows: (a) between‐group differences (i.e., ME/CFS and controls) are only observed during the second CPET and not the first, and (b) within‐group differences (i.e., first vs. second CPET performance) are only observed for patients with ME/CFS. Therefore, Stevens and colleagues (Stevens et al., 2018) suggest that the magnitude of CPET performance decrement between bouts can serve as an objective diagnostic marker for patients with ME/CFS, provide a better understanding of the underlying pathophysiology, quantify the degree of PEM‐induced disability impairment rating, as well as inform therapy and illness progression.

Recent studies have similarly adopted the 2‐day CPET protocol for the study of patients with multiple sclerosis (Hodges, Nielsen, & Baken, 2018) and sarcoidosis (Braam et al., 2013). Therefore, application of the 2‐day CPET protocol has been employed in several clinical populations characterized by fatigue and PEM (Bouquet et al., 2019; Braam et al., 2013; Campen, Rowe, & Visser, 2020; Hodges et al., 2018; Keller, Pryor, & Giloteaux, 2014; Lien et al., 2019; Nelson et al., 2019; Snell, Stevens, Davenport, & Van Ness, 2013; Vanness, Snell, & Stevens, 2007; Vermeulen, Kurk, Visser, Sluiter, & Scholte, 2010). A major assumption for the 2‐day CPET protocol is that measurements are both stable over time and sensitive to change. High test–retest reliability has been reported in healthy and chronic disease populations, as reviewed elsewhere (Balady et al., 2010); however, sensitivity to change has been questioned particularly among individuals with fatiguing illness (Heine, van den Akker, Verschuren, Visser‐Meily, & Kwakkel, 2015). Reliability of CPET and its responsiveness to change have not been thoroughly evaluated among individuals who may experience PEM (i.e., ME/CFS and multiple sclerosis), but would yield considerable insight into the utility of 2‐day CPET protocol and inform its interpretation.

Approximately 25%–32% of military veterans of Operations Desert Storm and Shield (1990–1991) are afflicted with a chronic multisymptom illness referred to as Gulf War Illness (GWI) (White et al., 2016) with persistent fatigue being a cardinal symptom. GWI and ME/CFS have several overlapping characteristics, including the lack of objective indicators, similar symptom profiles, and exercise‐induced symptom exacerbation (Lindheimer et al., 2020). Work from our laboratory and others has employed exercise testing in veterans with GWI as a stressor to elucidate underlying mechanisms of this illness (Broderick et al., 2011, 2013; Cook et al., 2003; Cook, Stegner, & Ellingson, 2010; Lindheimer et al., 2019; Rayhan, Raksit, et al., 2013; Rayhan, Stevens, et al., 2013; Smylie et al., 2013; Whistler et al., 2009). However, these studies have focused on a single exercise test and the 2‐day CPET protocol has not previously been studied in GWI. Therefore, the present study aims to address this literature gap by quantifying the repeatability of directly measured CPET parameters and establishing their thresholds of change in a clinical population with fatiguing illness (i.e., veterans with GWI) and controls. Parts of these results have previously been reported (Chen et al., 2017; Lindheimer et al., 2019).

## 2 METHODS

### 2.1 Participants

A total of 35 individuals provided their written informed consent to participate in this study, including 13 controls (GWI‐) and 22 veterans (GWI+) who met both the Centers for Disease Control and Prevention (Fukuda et al., 1994) and Kansas case definition for Gulf War Illness (Steele, 2000). In brief, case status requires deployment to the Gulf War theater of operations between August 8, 1990 and July 31, 1991 and endorsement of moderate‐to‐severe chronic symptoms in three or more of the following domains: fatigue, pain, neurological/cognitive/mood, skin, gastrointestinal, and respiratory. Symptom onset must have occurred during or after deployment and independent of comorbid conditions (i.e., diabetes, heart disease, stroke, lupus, multiple sclerosis, cancer, etc.). Control participants consisted of both nondeployed, otherwise healthy Gulf War veterans and nonmilitary civilians. Participants from either group were excluded from the study if they had any of the following: 1) absolute contraindications to exercise (American College of Sports Medicine, 2013; Fletcher et al., 2013), 2) organ failure, 3) chronic infections (e.g., HIV/AIDS, hepatitis B or C), 4) major neurologic diseases, 5) diseases requiring systemic treatment (e.g., systemic chemotherapy, radiation of brain, thorax, abdomen, or pelvis), 6) major endocrine diseases, 7) history of myocardial infarction, heart failure, or heart disease, or 8) morbid obesity (body mass index >40). All exclusions were verified in the electronic health record in cases where self‐report was uncertain. Physical activity was assessed using the short‐form International Physical Activity Questionnaire (IPAQ) to derive metabolic equivalent minutes per week (Craig et al., 2003). Fatigue severity and its impact on quality of life were assessed via the 9‐item Fatigue Severity Scale (FSS) with scores >36 constituting clinical fatigue (Krupp, LaRocca, Muir‐Nash, & Steinberg, 1989). Lastly, physical health‐related functioning was quantified from the veterans version of the Short Form 36 Health Survey (VR‐36) where a score of 50 is considered the U.S. average (Kazis, 2000; Kazis, Skinner, Ren, & Perlin, 1999; Ware et al.,^2007^). Study procedures were reviewed and approved by the VA New Jersey Health Care System Institutional Review Board (#01251). All participants provided informed consent according to the Declaration of Helsinki prior to testing.

### 2.2 Cardiopulmonary exercise testing

Participants performed two consecutive maximal effort CPETs, approximately 24 hr apart (±2 hr), on a cycle ergometer (Ergoline, Ergoselect 200) using a ramp protocol (15 watts∙min^−1^; 50–70 rpm) until volitional exhaustion. A clinical exercise physiologist supervised all CPETs and ensured participant safety. Heart rate and rhythm (Cosmed T12x; Rome, Italy), and oxygen saturation were monitored continuously. Blood pressure was manually auscultated approximately every 2 min during exercise and into recovery. Perceived exertion (Rating of Perceived Exertion; 6–20 scale) and breathlessness (Borg Breathlessness Scale; 0 – 10 scale) were measured each minute throughout exercise and at 2‐, 5‐, and 10 min of recovery. Pulmonary gas exchange and ventilation were measured breath‐by‐breath using an oronasal mask (V2 Series; Hans Rudolph, Shawnee, KS) connected to a metabolic cart (Cosmed Quark CPET; Rome, Italy). Testing was terminated when participants met maximal effort criteria or when they were no longer able to maintain pedaling frequency despite verbal encouragement. We defined valid effort as meeting two or more of the following criteria: 1) peak respiratory exchange ratio (RER) ≥ 1.1, 2) peak heart rate ≥ 85% of age‐predicted maximum, and/or 3) no change in the rate of oxygen consumption (V̇O_2_) < 2.1 ml∙min∙kg^−1^ over last minute (Taylor, Buskirk, & Henschel, 1955).

Raw breath‐by‐breath CPET data were visually inspected and averaged (15 breaths) for offline analyses (Robergs, Dwyer, & Astorino, 2010). Our a priori variables of interest were those directly measured during CPET and included V̇O_2_, rate of carbon dioxide production (V̇CO_2_), tidal volume (V_T_), breathing frequency (*f*_R_), heart rate (HR), work rate (WR), and rating of perceived exertion (RPE). Variables were reported at peak exercise and the ventilatory anaerobic threshold (VAT), except for RPE which was reported at peak exercise only. The VAT was determined by a clinical exercise physiologist using the modified V‐slope approach (Beaver, Wasserman, & Whipp, 1986).

### 2.3 Statistical analysis

Participant characteristics were compared with independent samples *t*‐tests (α < 0.05), Hedges’ *d* effect sizes, and 95% confidence intervals (95% CI) (Fritz, Morris, & Richler, 2012). To examine potential changes in CPET parameters from Day 1 to Day 2, we calculated a series of separate two‐way repeated measures ANOVAs with group (GWI + and GWI‐) as the between‐subjects factor and time (Day 1 and Day 2) as the within‐subjects factor. A significant group‐by‐time interaction (α < 0.05) would indicate differential changes in those with (GWI+) relative to without (GWI‐) GWI. Partial eta‐squared (η^2^_p_) effect sizes of 0.01, 0.06, and 0.14 suggest small, medium, and large effects, respectively (Cohen, 1988). Based on prior studies using the 2‐day CPET protocol in ME/CFS patients and those directly measured during CPET, we selected the following dependent variables: V̇O_2_, V̇CO_2_, V_T_, *f*_R_, HR, RPE, and WR. To aid in the interpretation of the results from the RM‐ANOVA models, we followed the recommendations of Lexell and Downham to assess the reliability of the 2‐day CPET protocol through a series of additional statistical analyses (Lexell & Downham, 2005). First, we examined test–retest reliability by calculating intraclass correlation coefficients (ICC) with a 95% CI using a two‐way mixed effect model for absolute agreement from single measures. Values less than 0.5 indicate poor reliability, values between 0.5 and 0.75 indicate moderate reliability, values between 0.75 and 0.9 indicate good reliability, and values greater than 0.90 indicate excellent reliability (Koo & Li, 2016). Second, to check for systematic bias and outliers, we used Bland‐Altman plots to compare the difference between CPET parameters on Day 1 and Day 2 (y‐axis) against the mean value of the parameter across both days (x‐axis) for each participant. For all CPET variables at peak and VAT, Bland‐Altman plots report biases and lower limit of agreements with 95% CIs as recommended by Bland and Altman (Bland & Altman, 1999). Third, to evaluate the degree to which a given CPET parameter would need to change in order to be considered clinically significant [i.e., exceeding the standard error of the measure (Dvir, 2015)], we calculated the smallest real difference (SRD) with 95% CI (Lexell & Downham, 2005). The SRD is estimated using the following formula: SRD = 1.96 × √*SEM* × √2, where *SEM* is the square root of the mean square error term from the RM‐ANOVA. To provide a unit independent index that could be more easily interpreted, we also calculated the SRD% by dividing the SRD by the mean of the Day 1 and Day 2 measures and multiplying by 100 (Lexell & Downham, 2005). For each of these steps, data analyses were split by group (GWI+ and GWI‐).

## 3 RESULTS

Of the 35 participants, 23 (GWI+ = 15 and GWI‐ = 8) met the criteria for a valid peak effort (i.e., RER, HR, and/or V̇O_2_) on both testing sessions. The distribution of effort criteria was similar between groups across visits and a breakdown is provided in Table 1. For excluded GWI + participants, valid effort was not achieved at both visits (*n* = 2), visit 1 only (*n* = 2), and visit 2 only (*n* = 3). For the GWI‐ participants excluded, valid effort was not achieved at both visits (*n* = 4) and visit 2 only (*n* = 1). No significant differences were observed for demographic or other participant characteristics (age, sex, body fat percentage, tobacco use, FSS, and VR‐36) between those providing valid effort on both days versus those that did not (all *p* > .05). The following results section focuses on those 23 participants meeting criteria for valid peak effort, but tables and figures for the full sample are also provided.

**Table 1:** Percentage of participants meeting individual criteria for valid peak effort on both visits (*valid peak) and for the entire sample (full sample)*

	GWI+				GWI‐			
	Visit 1		Visit 2		Visit 1		Visit 2	
	Valid peak (*n* = 15)	Full sample (*n* = 22)	Valid peak *(n* = 15)	Full sample (*n* = 22)	Valid peak (*n* = 8)	Full sample (*n* = 13)	Valid peak (*n* = 8)	Full sample (*n* = 13)
RER	53.3	50.0	73.3	59.1	87.5	61.5	87.5	61.5
HR	73.3	63.6	73.3	54.5	50.0	38.5	37.5	30.8
V̇O_2_	86.7	81.8	93.3	95.5	100.0	92.3	87.5	76.9
RPE*	33.3	36.4	33.3	31.8	50.0	38.5	50.0	30.8

Note

HR, heart rate; RER, respiratory exchange ratio; RPE, rating of perceived exertion; V̇O_2_, oxygen consumption.

Full sample—includes participants who did not meet valid peak effort criteria on one or both exercise tests

Valid peak—the restricted sample of participants who met valid peak effort criteria on both exercise tests

Rating of perceived exertion (RPE) was not used to judge whether participants provided a valid peak effort, but is included to provide further context to these results.

### 3.1 Participants

Participant characteristics including their age, sex, smoking history, body mass index, physical activity, fatigue severity, and physical health‐related functioning are provided for participants meeting valid peak effort criteria in Table 2 and the full sample in Table 3.

**Table 2:** Comparison of mean (*SD*) of participant characteristics between veterans with Gulf War Illness (GWI+) and controls (GWI‐) who met criteria for valid peak effort for both exercise tests

	GWI+ n = 15	GWI‐ n = 8	p	Hedges’ d (95% CI)
Age (years)	49.4 (6.1)	50.3 (6.0)	.72	−0.08 (−0.93. 0.78)
Sex (female | male)	2|13	2|6	NA	NA
Body mass index (kg/m^2^)	29.72 (3.18)	29.22 (6.41)	.84	0.11 (−0.75, 0.97)
Physical Activity (MET‐min·wk^−1^)	859.57 (682.9)	2,459.88 (1952.7)	.06	−1.23 (−2.16, −0.30)
Tobacco (pack‐years)	6.0 (12.2)	8.7 (9.7)	.59	−0.23 (−1.09, 0.63)
Fatigue Severity Scale (FSS) sum score	46.33 (17.6)	24.38 (13.6)	.002	1.49 (0.53, 2.45)
Physical Composite Score (VR‐36)	38.57 (10.0)	55.75 (10.7)	.001	−1.62 (−2.6, −0.64)

Note

Group comparisons were conducted with a series of independent samples *t*‐tests (α = 0.05)

Positive Hedges’ *d* effect sizes indicate larger values in GWI+, and negative effect sizes indicate larger values in GWI‐.

Abbreviations: CI, confidence interval; NA, Not applicable.

**Table 3:** Comparison of mean (*SD*) of participant characteristics between veterans with Gulf War Illness (GWI+; *n* = 22) and controls (GWI‐; *n* = 13)

	GWI + n = 22	GWI ‐ n = 13	p	Hedges’ d (95% CI)
Age (years)	49.2 (5.415)	53.00 (6.178)	.07	−0.65 (−1.35, 0.06)
Sex (female | male)	3|19	3|10	NA	NA
Body mass index (kg/m^2^)	25.88 (6.5)	23.54 (9.0)	.84	−0.08 (−0.76, 0.61)
Physical Activity (MET‐min·wk^−1^)	886.61 (835.5)	2,476.04 (1,860.14)	.01	−1.19 (−1.93, −0.45)
Tobacco (pack‐years)	8.1 (13.204)	6.1 (8.2)	.58	0.17 (−0.52, 0.86)
Fatigue Severity Scale (FSS) sum score	46.82 (13.0)	25.38 (13.6)	<.001	1.59 (0.81, 2.37)
Physical Composite Score (VR‐36)	37.6 (9.5)	54.0 (10.5)	<.001	−1.63 (−2.41, −0.84)

Note

Group comparisons were conducted with a series of independent samples *t*‐tests (α = 0.05)

Positive Hedges’ *d* effect sizes indicate larger values in GWI+, and negative effect sizes indicate larger values in GWI‐.

Abbreviations: CI, confidence interval; NA, Not applicable.

### 3.2 Repeated measures ANOVA

**Figure 1:**
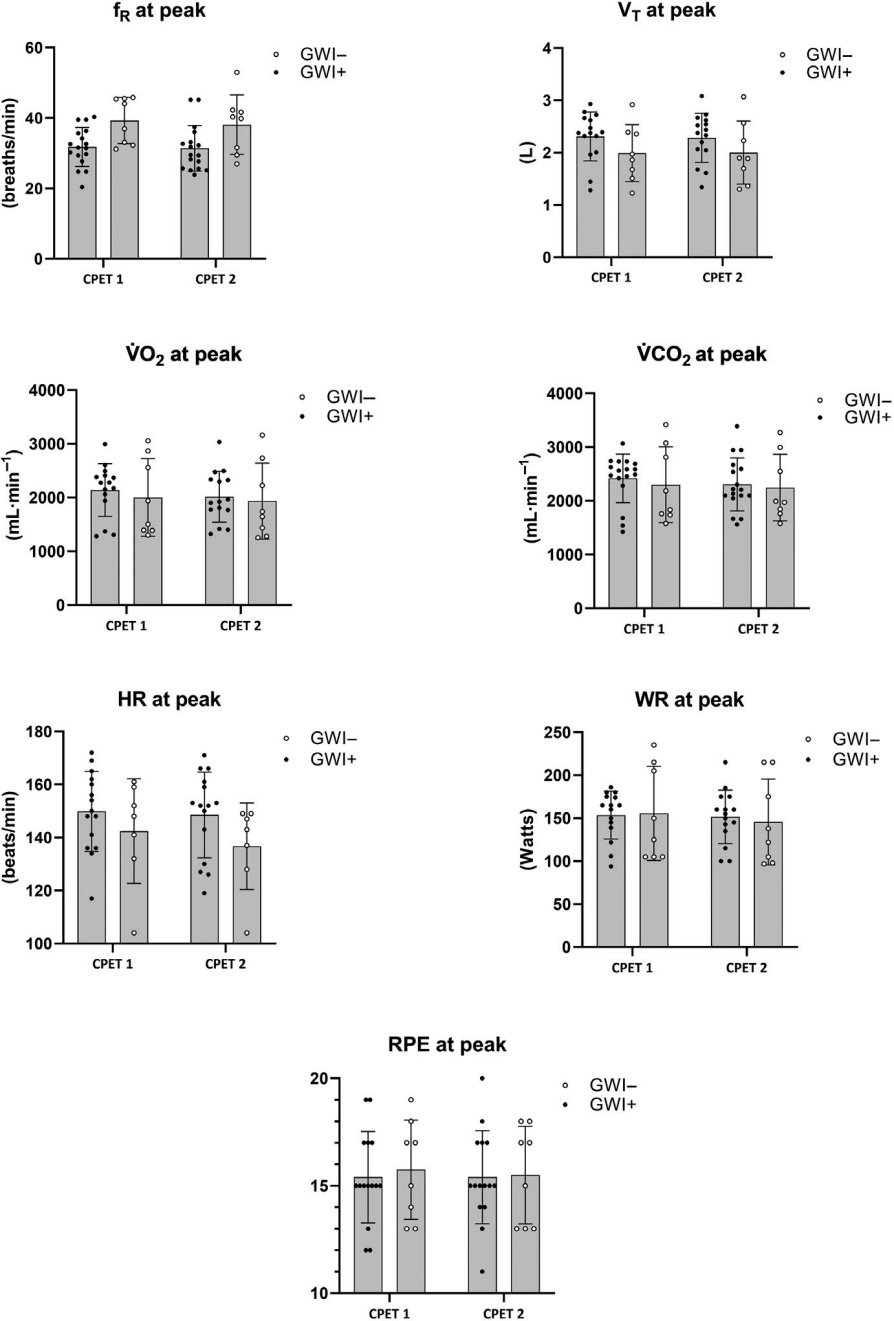
Mean (*SD*) CPET parameters for GWI + and GWI‐ at peak exercise restricted to participants who met valid peak effort criteria for both exercise tests. *f*_R_, respiratory frequency; HR, heart rate; RPE, rating of perceived exertion; VAT, ventilatory anaerobic threshold; V̇CO_2_, carbon dioxide production; V̇O_2_, oxygen consumption; V_T_, tidal volume; WR, work rate

**Figure 2:**
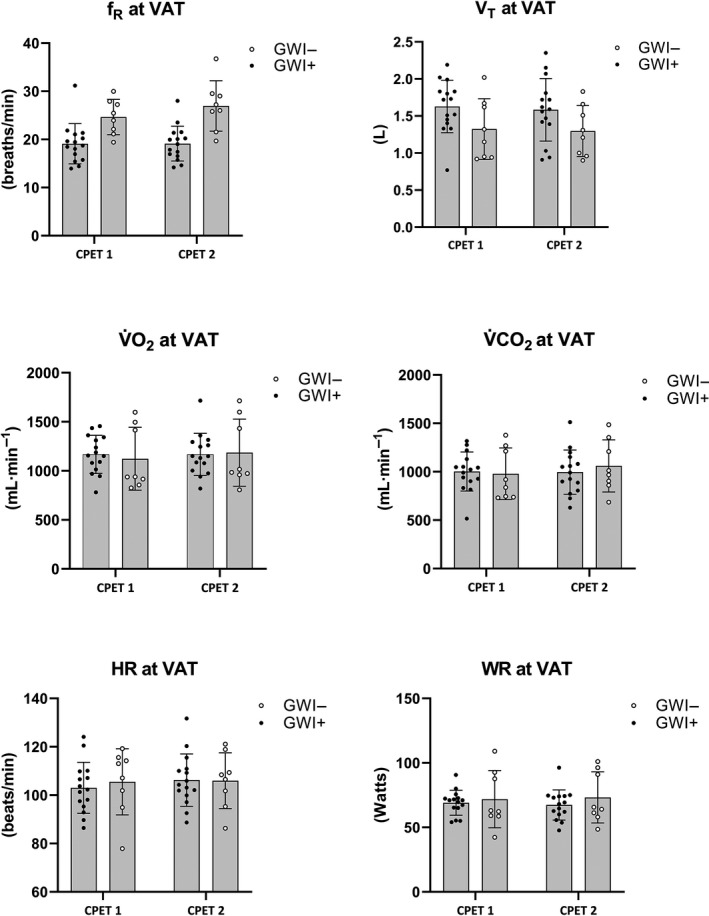
Mean (*SD*) CPET parameters for GWI + and GWI‐ at ventilatory anaerobic threshold restricted to participants who met valid peak effort criteria for both exercise tests. Note. *f*_R_, respiratory frequency; HR, heart rate; RPE, rating of perceived exertion; VAT, ventilatory anaerobic threshold; V̇CO_2_, carbon dioxide production; V̇O_2,_ oxygen consumption; V_T,_ tidal volume; WR, work rate

**Figure 3:**
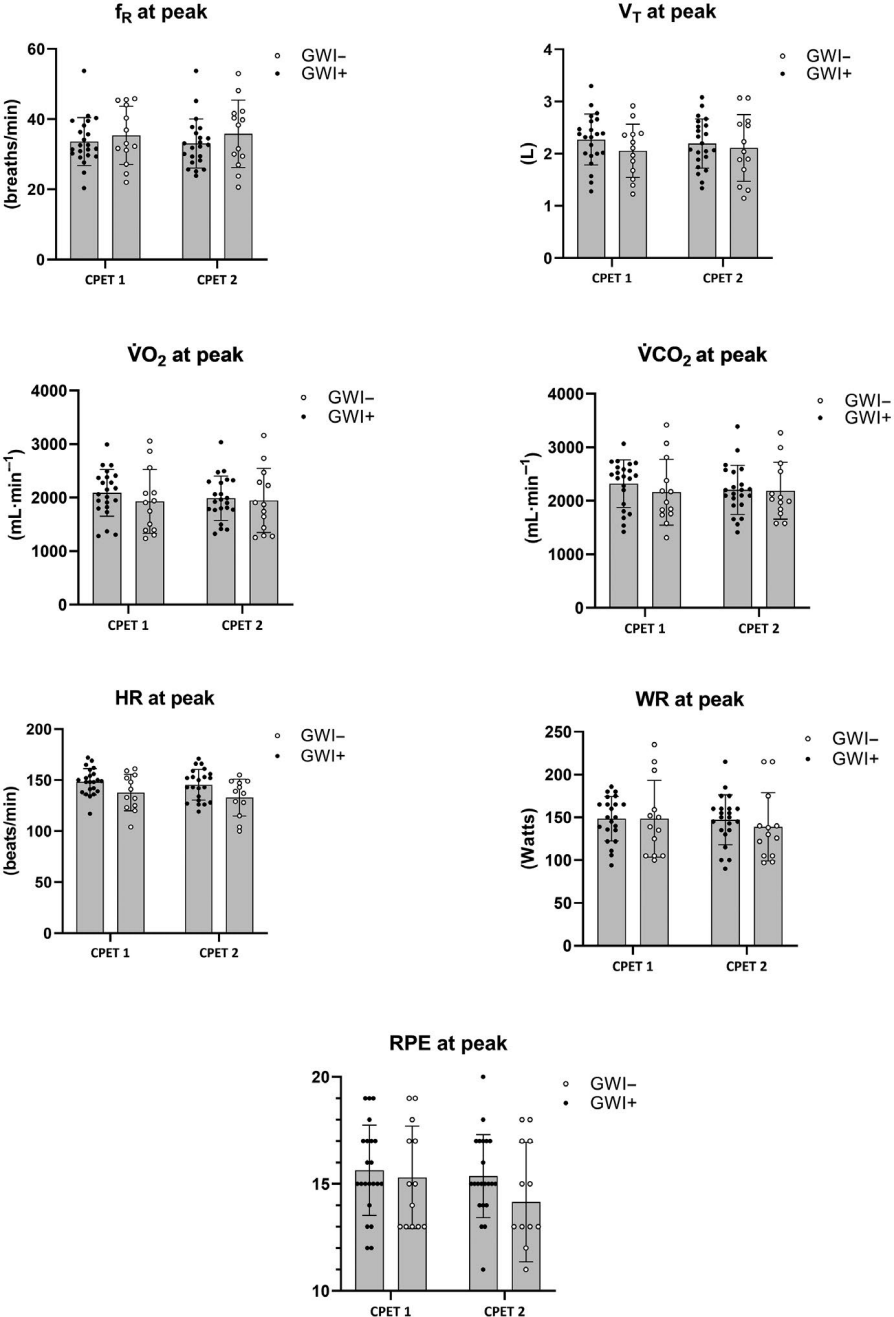
Mean (*SD*) CPET parameters for the full sample of GWI + and GWI‐ at peak exercise. *f*_R_, respiratory frequency; HR, heart rate; RPE, rating of perceived exertion; VAT, ventilatory anaerobic threshold; V̇CO_2_, carbon dioxide production; V̇O_2,_ oxygen consumption; V_T,_ tidal volume; WR, work rate

**Figure 4:**
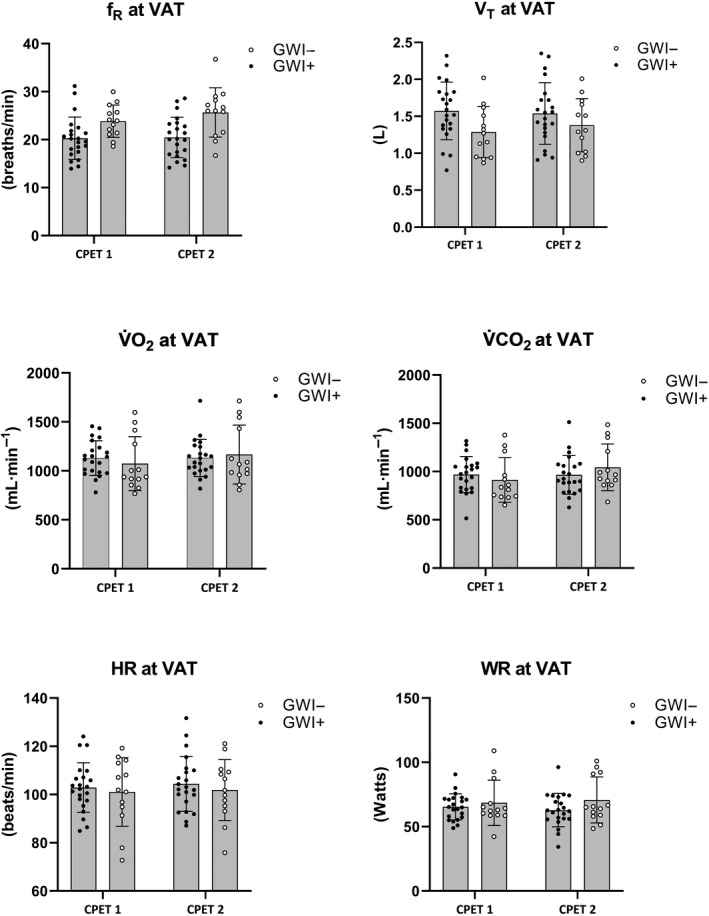
Mean (*SD*) CPET parameters for the full sample of GWI + and GWI‐ at ventilatory anaerobic threshold. Note. *f*_R_, respiratory frequency; HR, heart rate; RPE, rating of perceived exertion; VAT, ventilatory anaerobic threshold; V̇CO_2_, carbon dioxide production; V̇O_2,_ oxygen consumption; V_T,_ tidal volume; WR, work rate

Results of RM‐ANOVA are summarized in Table 4 for participants meeting valid peak effort criteria and Table 5 for the full sample. We did not observe significant group‐by‐time interactions at VAT or peak for any CPET parameters (all *p* > .05). Effect sizes for these tests ranged from small (η^2^_p_ = 0.001) to moderate (η^2^_p_ = 0.13). We did observe a significant and large group effect for peak *f*_R_ (*F* = 8.75, *p* = .007, η^2^_p_ = 0.29) and time effect for peak WR (*F* = 5.46, *p* = .03, η^2^_p_ = 0.21). Mean (*SD*) changes across CPET 1 and 2 are illustrated in Figures 1, 2 for participants meeting valid peak effort criteria and Figures 3, 4 for the full sample.

**Table 4:** Repeated measures ANOVA of peak CPET parameters across two maximal exercise tests restricted to veterans with Gulf War Illness (GWI+; *n* = 15) and controls (GWI‐; *n* = 8) who met criteria for valid peak effort on both exercise tests

	Group effect			Time effect			Group × Time effect		
	*F*	*p*	ES	*F*	*p*	ES	*F*	*p*	ES
V̇O_2_ (mL·min^−1^)	0.20	.66	0.01	4.24	.05	0.17	0.37	.55	0.02
V̇O_2_ (mL·min^−1^) at VAT	0.02	.90	0.001	0.97	.34	0.04	0.98	.33	0.04
V̇CO_2_ (mL·min^−1^)	0.06	.81	0.003	2.71	.12	0.11	0.30	.59	0.01
V̇CO_2_ (mL·min^−1^) at VAT	0.05	.83	0.002	0.92	.35	0.04	1.23	.28	0.06
V_T_ (L)	1.92	.18	0.08	0.02	.89	0.001	0.26	.62	0.01
V_T_ (L) at VAT	3.34	.08	0.14	0.52	.48	0.02	0.03	.86	0.001
*f*_R_ (breaths/min)	8.75	**.007**	0.29	0.84	.37	0.04	0.02	.89	0.001
*f*_R_ (breaths/min) at VAT	15.77	0	<0.001	3.28	.08	0.13	3.09	.08	0.13
HR (beats/min)	1.76	.20	0.08	4.36	.05	0.18	1.83	.19	0.08
HR (beats/min) at VAT	0.05	.82	0.002	2.14	.16	0.09	1.18	.29	0.05
Work Rate (watts)	0.01	.91	0.001	5.46	**.03**	0.21	2.22	.15	0.10
Work Rate (watts) at VAT	0.49	.49	0.02	0.004	.95	0.0002	0.41	.53	0.02
RPE	0.06	.81	0.003	0.22	.65	0.01	0.22	.65	0.01

Note

Effect sizes reported as partial eta‐squared.

Abbreviations: ES, effect size; F, F ratio; *f*_R_, respiratory frequency; HR, heart rate; ICC, Intraclass correlation; RPE, rating of perceived exertion; VAT, ventilatory anaerobic threshold; V̇CO_2_, carbon dioxide production; V̇O_2,_ oxygen consumption; V_T,_ tidal volume.

^a^ Data missing for one control participant.

**Table 5:** Repeated measures ANOVA of peak CPET parameters across two maximal exercise tests in the full sample of veterans with Gulf War Illness (GWI+; *n* = 22) and controls (GWI‐; *n* = 13)

	Group effect			Time effect			Group × Time effect		
	*F*	*p*	ES	*F*	*p*	ES	*F*	*p*	ES
V̇O_2_ (mL·min^−1^)	0.35	.56	0.01	0.95	.34	0.03	1.92	.18	0.06
V̇O_2_ (mL·min^−1^) at VAT	0.02	.89	0.001	3.51	.07	0.10	3.29	.08	0.09
V̇CO_2_ (mL·min^−1^)	0.28	.60	0.01	0.84	.37	0.3	2.34	.14	0.07
V̇CO_2_ (mL·min^−1^) at VAT	0.03	.87	0.001	4.14	.05	0.11	4.68	.04	0.12
V_T_ (L)	0.73	.40	0.02	0.05	.82	0.002	2.07	.16	0.06
V_T_ (L) at VAT	3.12	.09	0.09	0.32	.58	0.01	1.54	.22	0.04
*f*_R_ (breaths/min)	0.80	.38	0.02	0.003	.96	0.00008	0.26	.61	0.01
*f*_R_ (breaths/min) at VAT	9.63	.004	0.23	3.50	.07	0.10	2.40	.13	0.07
HR (beats/min)	5.07	.31	0.14	3.77	.06	0.11	0.22	.65	0.01
HR (beats/min) at VAT	0.30	.58	0.01	1.02	.32	0.03	0.10	.75	0.00
Work Rate (watts)	0.12	.73	0.004	7.64	.009	0.19	4.55	.04	0.12
Work Rate (watts) at VAT	1.50	.23	0.04	0.01	.94	0.0002	1.38	.25	0.04
RPE	1.09	.30	0.03	6.53	.02	0.17	2.49	.12	0.07

Note

Effect sizes reported as partial eta‐squared.

Abbreviations: ES, effect size; F, F ratio; *f*_R_, respiratory frequency; HR, heart rate; ICC, Intraclass correlation; RPE, rating of perceived exertion; VAT, ventilatory anaerobic threshold; V̇CO_2_, carbon dioxide production; V̇O_2,_ oxygen consumption; V_T,_ tidal volume.

^a^ Data missing for one control participant.

### 3.3 Test–retest reliability, bias, and clinically important change

**Figure 5:**
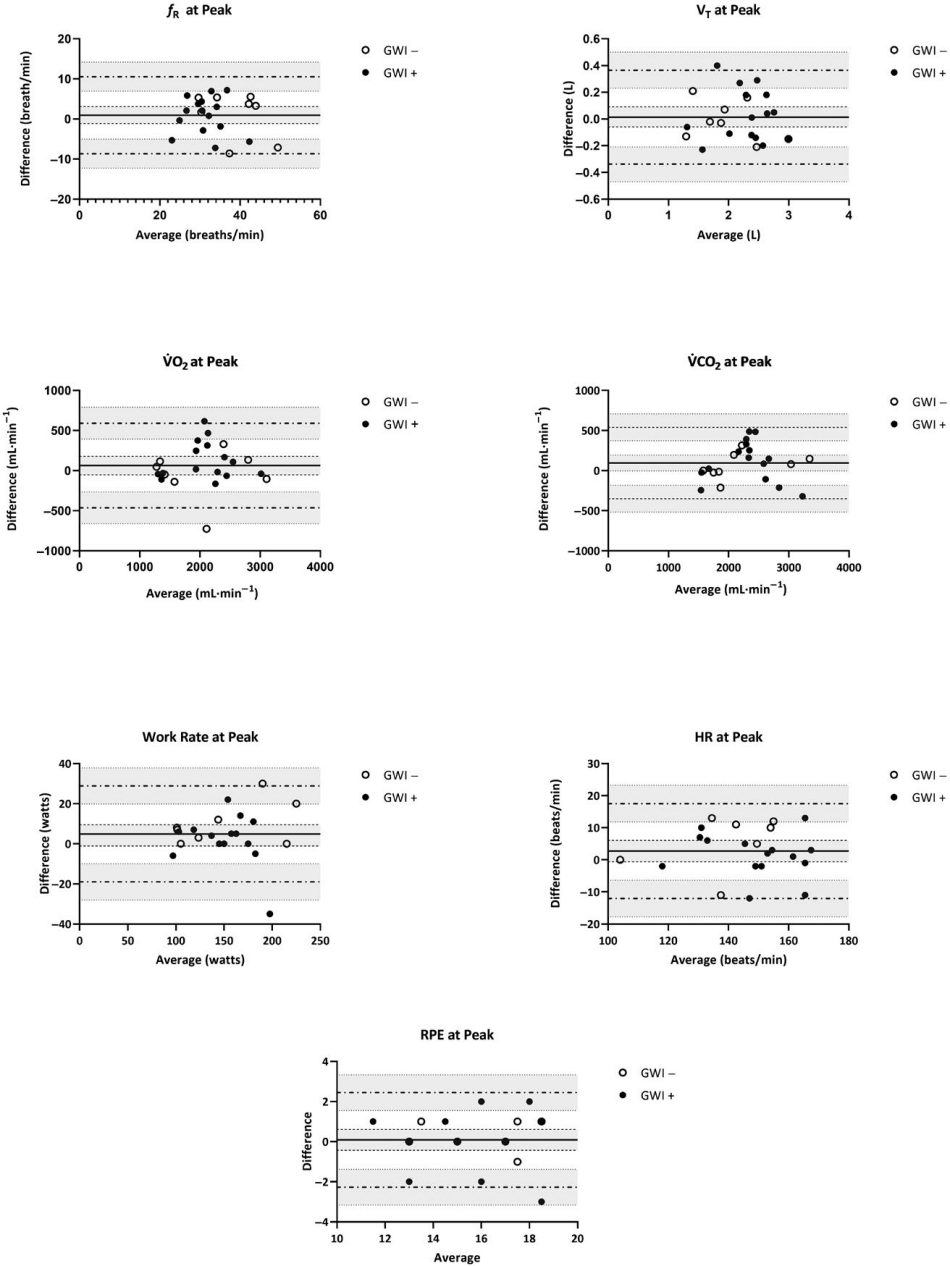
*5*Bland‐Altman plots of CPET parameters at peak exercise across two maximal exercise tests restricted to GWI + and GWI‐ who met valid peak effort criteria for both exercise tests: difference (CPET 1 – CPET 2) versus average values measured at CPET 1 and CPET 2. For each parameter (*f_R_*, V_T_, V̇O_2_, V̇CO_2_, HR, WR, and RPE), the bias (*solid horizontal line*) and 95% limits of agreement (*dashed horizontal lines*) are plotted with their corresponding 95% confidence intervals (*shaded regions*). Note. *f*_R_, respiratory frequency; HR, heart rate; RPE, rating of perceived exertion; VAT, ventilatory anaerobic threshold; V̇CO_2_, carbon dioxide production; V̇O_2,_ oxygen consumption; V_T,_ tidal volume

**Figure 6:**
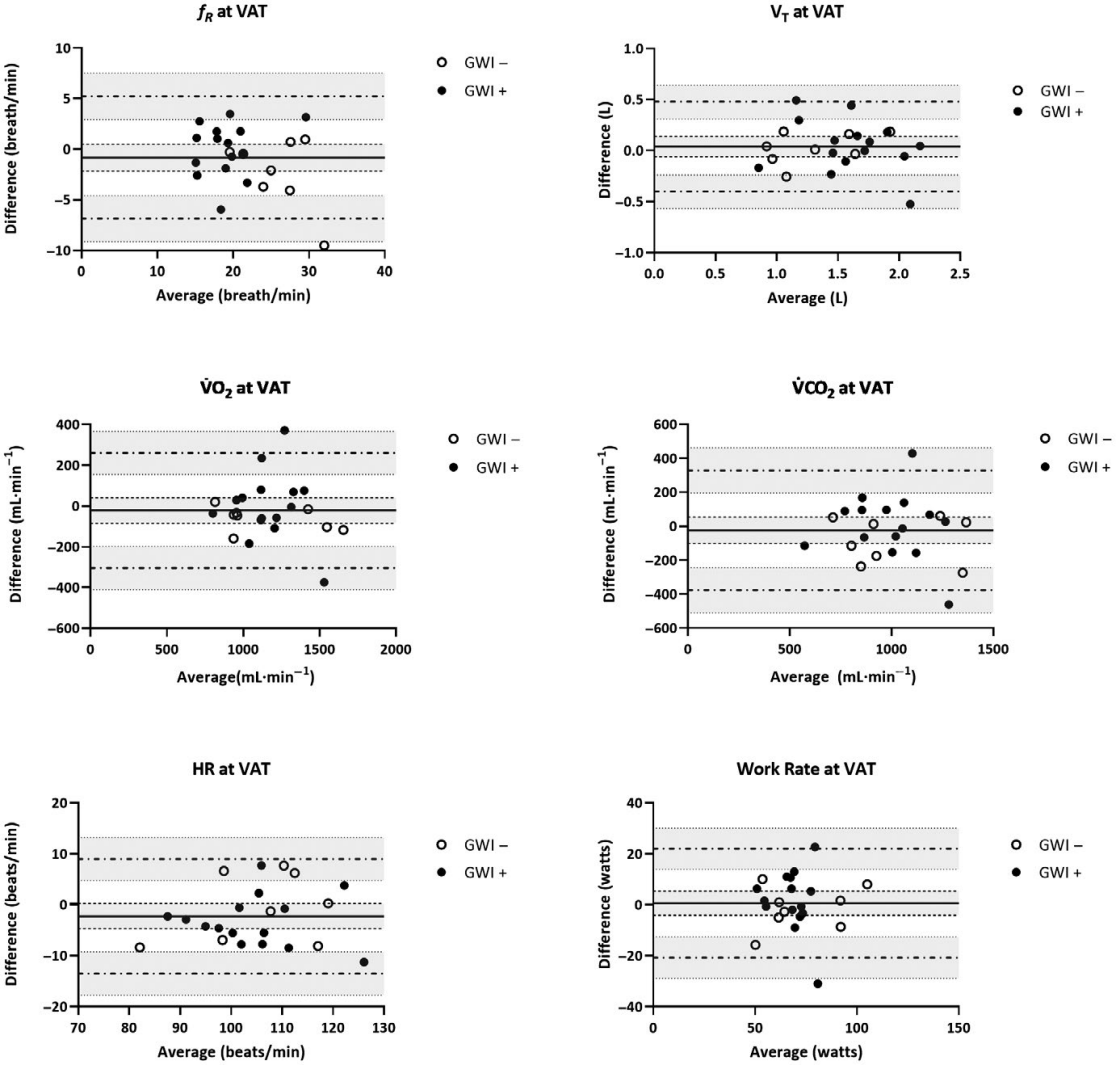
Bland‐Altman plots of CPET parameters at the ventilatory anaerobic threshold (VAT) across two maximal exercise tests restricted to GWI + and GWI‐ who met valid peak effort criteria for both exercise tests: difference (CPET 1 – CPET 2) versus average values measured at CPET 1 and CPET 2. For each parameter (*f_R_*, V_T_, V̇O_2_, V̇CO_2_, HR, and WR), the bias (*solid horizontal line*) and 95% limits of agreement (*dashed horizontal lines*) are plotted with their corresponding 95% confidence intervals (*shaded regions*). Note. *f*_R_, respiratory frequency; HR, heart rate; RPE, rating of perceived exertion; VAT, ventilatory anaerobic threshold; V̇CO_2_, carbon dioxide production; V̇O_2,_ oxygen consumption; V_T,_ tidal volume

**Figure 7:**
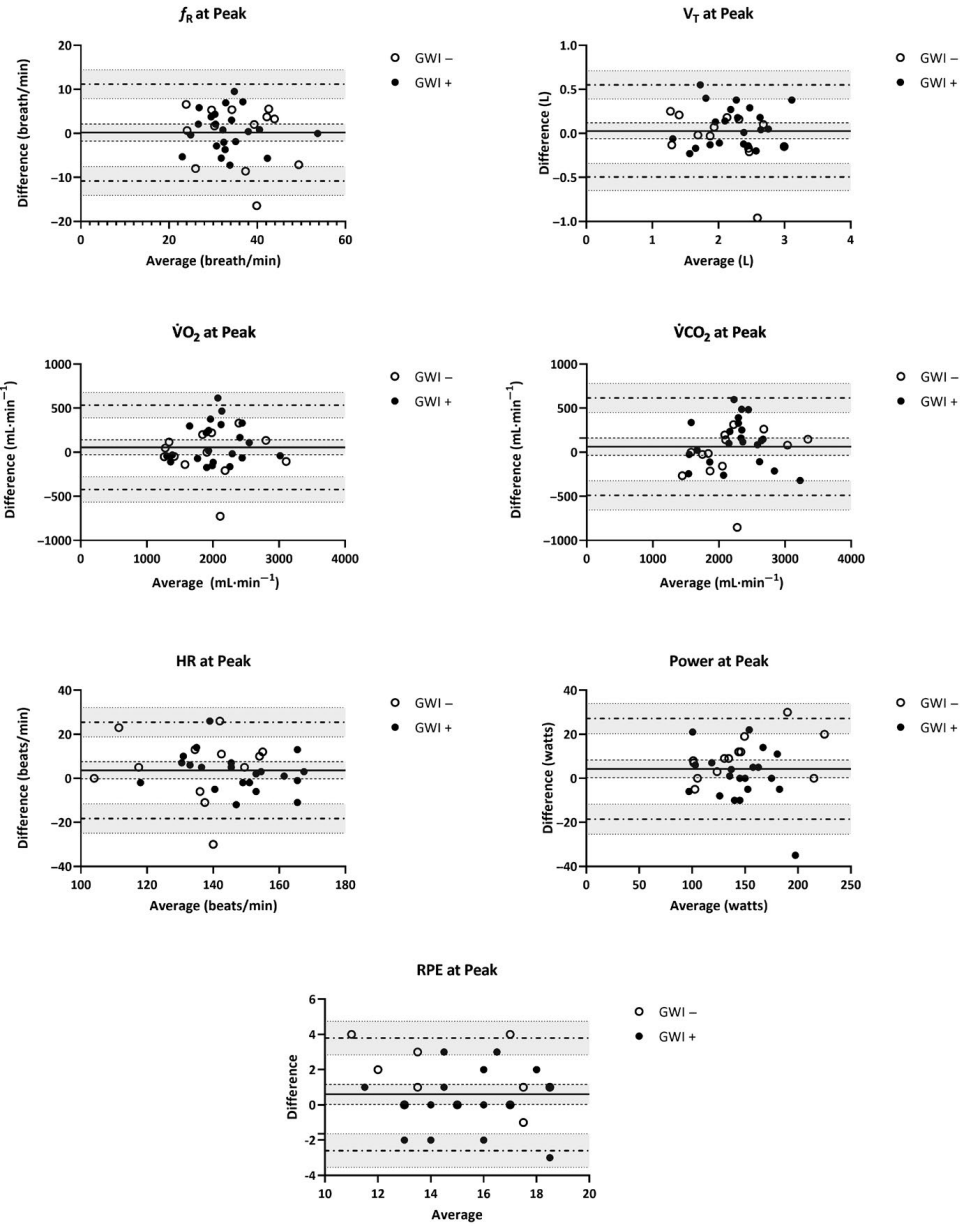
Bland‐Altman plots of CPET parameters at peak exercise across two maximal exercise tests for the full sample of GWI + and GWI‐ veterans: difference (CPET 1 – CPET 2) versus average values measured at CPET 1 and CPET 2. For each parameter (*f_R_*, V_T_, V̇O_2_, V̇CO_2_, HR, WR, and RPE), the bias (*solid horizontal line*) and 95% limits of agreement (*dashed horizontal lines*) are plotted with their corresponding 95% confidence intervals (*shaded regions*)

**Figure 8:**
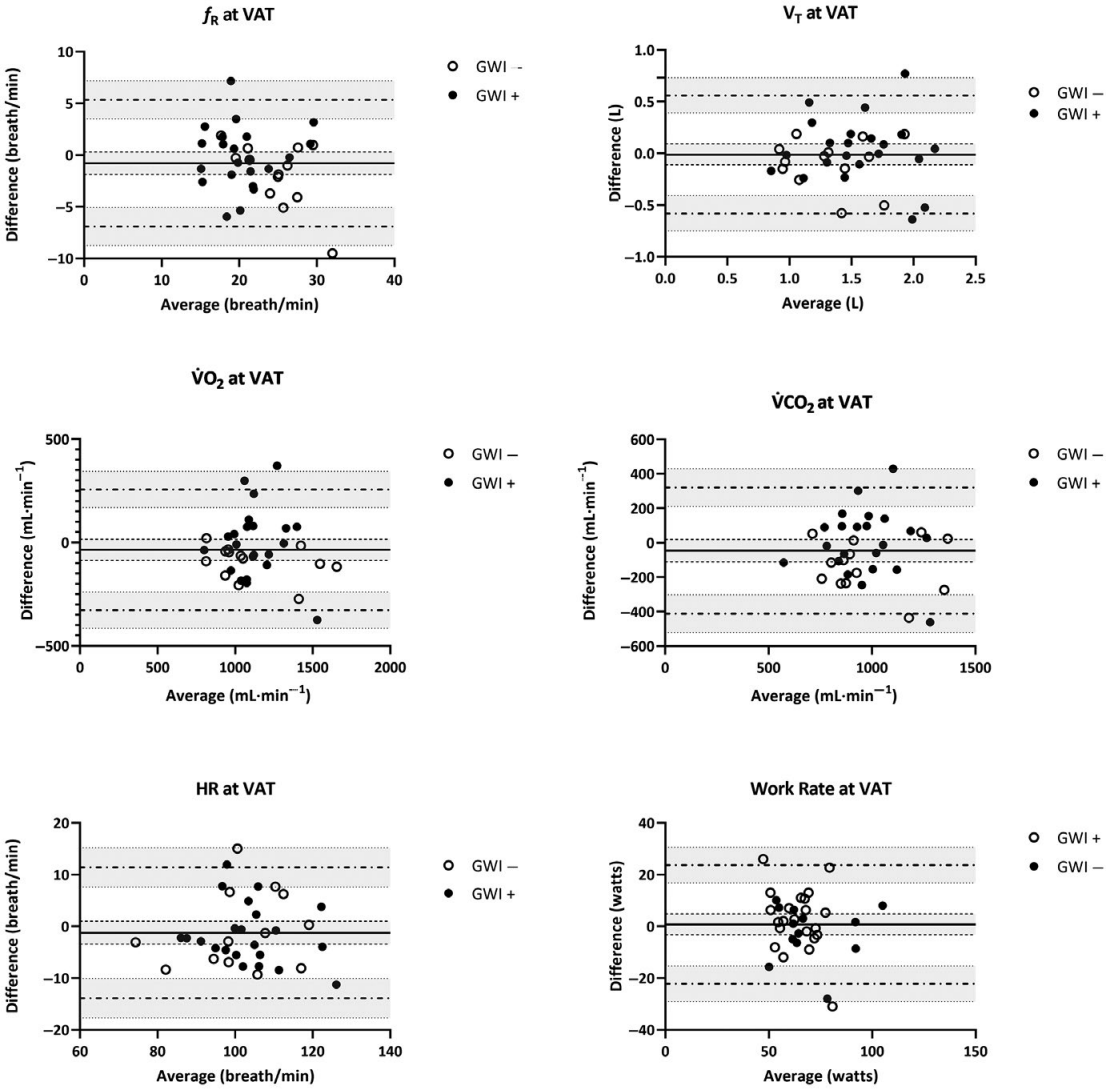
Bland‐Altman plots of CPET parameters at VAT across two maximal exercise tests for the full sample of GWI+ and GWI‐ veterans: difference (CPET 1 – CPET 2) versus average values measured at CPET 1 and CPET 2. For each parameter (*f_R_*, V_T_, V̇O_2_, V̇CO_2_, HR, WR, and RPE), the bias (*solid horizontal line*) and 95% limits of agreement (*dashed horizontal lines*) are plotted with their corresponding 95% confidence intervals (*shaded regions*)

Results for ICCs (95% CI) examining test–retest reliability across tests 1 and 2 for each group's CPET parameters are provided in Table 6 for participants meeting valid peak effort criteria and Table 7 for the full sample. With the exception of WR at VAT for the GWI + group (0.37; 95% CI: −0.17, 0.74), ICCs for both groups ranged from moderate to excellent. Average ICCs across CPET parameters for each group indicated lower test–retest reliability in the GWI + group (0.76) than the GWI‐ group (0.90). Using Bland‐Altman plots to check for systematic bias and outliers, estimated values for bias (95% CI) as well as lower (95% CI) and upper (95% CI) limits of agreement for CPET parameters at VAT and peak are reported in Table 8. Bland‐Altman plots are shown in Figures 5, 6 for participants meeting valid peak effort criteria and Table 9 and Figures 7, 8 for the full sample. Absolute SRD (95% CI) and SRD% values for GWI + and GWI‐ groups are presented in Table 10 for participants meeting valid peak effort criteria and Table 11 for the full sample.

**Table 6:** Test–retest reliability of CPET parameters across two maximal exercise tests restricted to veterans with Gulf War Illness (GWI+) and controls (GWI‐) who met valid peak effort criteria for both exercise tests

	GWI+ (*n* = 15)		GWI‐ (*n* = 8)	
	ICC	95% CI	ICC	**95% CI**
V̇O_2_ (mL·min^−1^)	0.86	0.61, 0.95	0.97	0.89, 0.99
V̇O_2_ (mL·min^−1^) at VAT	0.66	0.23, 0.87	0.97	0.65, 0.99
V̇CO_2_ (mL·min^−1^)	0.84	0.60, 0.94	0.97	0.89, 0.99
V̇CO_2_ (mL·min^−1^) at VAT	0.60	0.12, 0.84	0.85	0.42, 0.97
V_T_ (L)	0.92	0.77, 0.91	0.97	0.86, 0.99
V_T_ (L) at VAT	0.78	0.48, 0.92	0.92	0.68, 0.98
*f*_R_ (breaths·min^−1^)	0.65	0.22, 0.87	0.73	0.12, 0.94
*f*_R_ (breaths·min^−1^) at VAT	0.78	0.46, 0.92	0.64	0.04, 0.91
HR (beats·min^−1^)	0.91	0.76, 0.97	0.85^a^	0.38, 0.97
HR (beats·min^−1^) at VAT	0.86	0.55, 0.95	0.87	0.47, 0.97
Work Rate (watts)	0.91	0.77, 0.97	0.97	0.69, 0.99
Work Rate (watts) at VAT	0.37	−0.17, 0.74	0.93	0.68, 0.98
RPE	0.80	0.49, 0.93	0.95	0.80, 0.99

Note

ICCs and 95% CI calculated with two‐way mixed effect model for absolute agreement from single measures.

Abbreviations: CI, Confidence interval; *f*_R_, respiratory frequency; HR, heart rate; ICC, Intraclass correlation; RPE, rating of perceived exertion; VAT, ventilatory anaerobic threshold; V̇CO_2_, carbon dioxide production; V̇O_2,_ oxygen consumption; V_T,_ tidal volume.

^a^ Data missing for one participant.

**Table 7:** Test–retest reliability of peak CPET parameters across two maximal exercise tests in veterans with Gulf War Illness (GWI+) and controls (GWI‐)

	GWI+ (*n* = 22)		GWI‐ (*n* = 13)	
	ICC	95% CI	ICC	**95% CI**
V̇O_2_ (mL·min^−1^)	0.84	0.64, 0.93	0.91	0.73, 0.97
V̇O_2_ (mL·min^−1^) at VAT	0.58	0.22, 0.80	0.92	0.33, 0.98
V̇CO_2_ (mL·min^−1^)	0.81	0.58, 0.92	0.87	0.62, 0.96
V̇CO_2_ (mL·min^−1^) at VAT	0.52	0.12, 0.77	0.71	0.12, 0.91
V_T_ (L)	0.88	0.74, 0.95	0.86	0.61, 0.96
V_T_ (L) at VAT	0.71	0.41, 0.87	0.76	0.40, 0.92
*f*_R_ (breaths·min^−1^)	0.79	0.55, 0.91	0.70	0.25, 0.90
*f*_R_ (breaths·min^−1^) at VAT	0.76	0.51, 0.89	0.69	0.24, 0.90
HR (beats·min^−1^)	0.81	0.59, 0.91	0.64^a^	0.16, 0.88
HR (beats·min^−1^) at VAT	0.85	0.68, 0.93	0.85	0.58, 0.95
Work Rate (watts)	0.91	0.80, 0.96	0.95	0.62, 0.99
Work Rate (watts) at VAT	0.46	0.06, 0.73	0.82	0.52, 0.94
RPE	0.70	0.41, 0.86	0.74	0.27, 0.92

Note

ICCs and 95% CI calculated with two‐way mixed effect model for absolute agreement from single measures.

Abbreviations: CI, Confidence interval; *f*_R_, respiratory frequency; HR, heart rate; ICC, Intraclass correlation; RPE, rating of perceived exertion; VAT, ventilatory anaerobic threshold; V̇CO_2,_ carbon dioxide production; V̇O_2,_ oxygen consumption; V_T,_ tidal volume.

^a^ Data missing for one participant.

**Table 8:** Bland‐Altman bias (95% CI) and limits of agreement (95% CI) for CPET parameters across two maximal exercise tests restricted to veterans with Gulf War Illness (GWI+) and controls (GWI‐) who met valid peak effort criteria for both exercise tests

	Bias (95% CI)	Lower Limit (95% CI)	Upper Limit (95% CI)
V̇O_2_ (mL·min^−1^)	63.48 (−52.93, 179.89)	−464.10 (−663.16, −265.04)	591.10 (392.04, 790.16)
V̇O_2_ (mL·min^−1^) at VAT	−21.53 (−83.84, 40.78)	−303.90 (−410.46, −197.34)	260.80 (154.24, 367.36)
V̇CO_2_ (mL·min^−1^)	94.61 (−3.60, 192.82)	−350.40 (−518.33, −182.47)	539.60 (371.67, 707.53)
V̇CO_2_ (mL·min^−1^) at VAT	−24.28, (−102.16, 53.60)	−377.30 (−510.48, −244.12)	328.70 (195.52, 461.88)
V_T_ (L)	0.01 (−0.06,0.09)	0.34 (−0.47, −0.21)	0.36 (0.23, 0.50)
V_T_ (L) at VAT	0.04 (−0.06, 0.14)	−0.40 (−0.57, −0.24)	0.48 (0.31, 0.64)
*f*_R_ (breaths·min^−1^)	0.96 (−1.16, 3.07)	−8.64 (−12.26, −5.02)	10.56 (6.94, 14.18)
*f*_R_ (breaths·min^−1^) at VAT	−0.82 (−2.16, 0.51)	−6.87 (−9.15, −4.59)	5.23 (2.94, 7.51)
HR (beats·min^−1^)^a^	2.73 (−0.54, 5.99)	−12.07 (−17.65, −6.49)	17.52, (11.94, 23.10)
HR (beats·min^−1^) at VAT	−2.24 (−4.72, 0.24)	−13.48 (−17.72, −9.24)	8.99 (4.75, 13.23)
Work Rate (watts)	4.19 (−1.08,9.47)	19.00 (−28.02, −9.98)	28.82 (19.80, 37.84)
Work Rate (watts) at VAT	0.63 (−4.09, 5.35)	−20.77 (−28.84, −12.70)	22.03 (13.96, 30.10)
RPE	0.09 (−0.43, 0.61)	−2.27 (−3.16, −1.38)	2.44 (1.55, 3.33)

Note

Abbreviations: CI, Confidence interval; *f*_R_, respiratory frequency; HR, heart rate; RPE, rating of perceived exertion; VAT, ventilatory anaerobic threshold; V̇CO_2_, carbon dioxide production; V̇O_2,_ oxygen consumption; V_T,_ tidal volume.

^a^ Data missing for one control participant.

**Table 9:** Bland‐Altman bias (95% CI) and limits of agreement (95% CI) for CPET parameters across two maximal exercise tests in veterans with Gulf War Illness (GWI+) and controls (GWI‐)

	Bias (95% CI)	Lower Limit (95% CI)	Upper Limit (95% CI)
V̇O_2_ (mL·min^−1^)	56.18 (−27.74, 140.10)	−422.60 (−566.10, −279.10)	534.90 (391.40, 678.40)
V̇O_2_ (mL·min^−1^) at VAT	−35.50 (−86.65, 15.65)	−327.40 (−414.86, −239.94)	256.40 (168.94, 343.86)
V̇CO_2_ (mL·min^−1^)	63.29 (−33.55, 160.13)	−489.30 (−654.89, −323.71)	615.90 (450.31, 781.49)
V̇CO_2_ (mL·min^−1^) at VAT	−45.90 (−110.03, 18.23)	−411.80 (−521.47, −302.13)	320.00(210.33, 429.67)
V_T_ (L)	0.03 (−0.06, 0.12)	−0.49 (−0.65, −0.34)	0.55 (0.39, 0.71)
V_T_ (L) at VAT	−0.01 (−0.11, 0.09)	−0.58 (−0.75, −0.41)	0.56 (0.39, 0.73)
*f*_R_ (breaths·min^−1^)	0.18 (−1.74, 2.11)	−10.81 (−14.10, −7.52)	11.17 (7.88, 14.46)
*f*_R_ (breaths·min^−1^) at VAT	−0.79 (−1.86, 0.29)	−6.91 (−8.75, −5.08)	5.34 (3.50, 7.17)
HR (beats·min^−1^)^a^	3.65 (−0.24, 7.54)	−18.22 (24.87, −11.57)	25.51 (18.86, 32.16)
HR (beats·min^−1^) at VAT	−1.25 (−3.46, 0.97)	−13.89 (−17.68, −10.10)	11.40 (7.61, 15.19)
Work Rate (watts)	4.31 (0.31, 8.32)	−18.55 (−25.40, −11.70)	27.18 (20.33, 34.03)
Work Rate (watts) at VAT	0.78 (−3.24, 4.80)	−22.14 (−29.01, −15.27)	23.70 (16.83, 30.57)
RPE	0.60 (0.04, 1.16)	−2.60 (−3.55, −1.64)	3.80 (2.84, 4.75)

Abbreviations: CI, Confidence interval; *f*_R_, respiratory frequency; HR, heart rate; RPE, rating of perceived exertion; VAT, ventilatory anaerobic threshold; V̇CO_2,_ carbon dioxide production; V̇O_2,_ oxygen consumption; V_T,_ tidal volume.

^a^ Data missing for one participant.

**Table 10:** Smallest real difference (SRD) of CPET parameters across two maximal exercise tests restricted to veterans with Gulf War Illness (GWI+) and controls (GWI‐) who met valid peak effort criteria for both exercise tests

	GWI+ (*n* = 15)			GWI‐ (*n* = 8)		
	SRD	95% CI	SRD%	SRD	95% CI	SRD%
V̇O_2_ (mL·min^−1^)	454.53	331.29, 577.76	21.86	312.51	245.31, 379.71	15.87
V̇O_2_ (mL·min^−1^) at VAT	335.08	334.91, 335.25	28.69	115.84	53.62, 178.07	10.04
V̇CO_2_ (mL·min^−1^)	505.52	395.85, 615.19	21.70	293.32	238.59, 348.04	12.91
V̇CO_2_ (mL·min^−1^) at VAT	385.75	379.72, 391.78	38.60	264.84	183.73, 345.94	25.98
V_T_ (L)	0.38	0.35, 0.41	16.58	0.29	0.28, 0.30	14.52
V_T_ (L) at VAT	0.48	0.43, 0.52	29.81	0.28	0.25, 0.30	21.08
*f*_R_ (breaths·min^−1^)	9.01	8.16, 9.87	28.77	11.18	10.02, 12.34	28.88
*f*_R_ (breaths·min^−1^) at VAT	5.17	5.14, 5.20	27.05	6.79	4.49, 9.09	26.33
HR (beats·min^−1^)	13.08	11.84, 14.32	8.76	17.12	11.31, 22.93	12.26
HR (beats·min^−1^) at VAT	9.91	6.72, 13.09	9.47	13.31	12.84, 13.77	12.59
Work Rate (watts)	24.44	22.24, 26.64	16.01	20.48	10.53, 30.43	13.59
Work Rate (watts) at VAT	23.73	22.02, 25.44	34.77	16.62	15.22, 18.03	22.92
RPE	2.76	2.76, 2.76	17.95	1.38	1.13, 1.63	8.84

Note

SRD = 1.96 X √*SEM* X √2

*SEM* = √Mean Square Error from Repeated Measures Analysis of Variance.

95% confidence interval = SRD±mean difference between test 1 and test 2

SRD% = (SRD/Average of test 1 and test 2) × 100.

Abbreviations: CI, Confidence interval; *f*_R_, respiratory frequency; HR, heart rate; RPE, rating of perceived exertion; *SEM*, standard error of the measure; SRD, smallest real difference; VAT, ventilatory anaerobic threshold; V̇CO_2_, carbon dioxide production; V̇O_2,_ oxygen consumption; V_T,_ tidal volume.

^a^ Data missing for one participant.

**Table 11:** Smallest real difference (SRD) of peak CPET parameters across two maximal exercise tests in veterans with Gulf War Illness (GWI+) and controls (GWI‐)

	GWI+ (*n* = 22)			GWI‐ (*n* = 13)		
	SRD	95% CI	SRD%	SRD	95% CI	SRD%
V̇O_2_ (mL·min^−1^)	444.19	344.63, 543.74	21.79	514.59	497.36, 531.82	26.55
V̇O_2_ (mL·min^−1^) at VAT	332.32	330.80, 333.83	29.38	158.12	65.10, 251.14	14.11
V̇CO_2_ (mL·min^−1^)	507.11	388.97, 625.25	22.40	594.41	564.89, 623.94	27.33
V̇CO_2_ (mL·min^−1^) at VAT	375.47	371.54, 379.39	38.80	289.17	158.94, 419.40	29.55
V_T_ (L)	0.45	0.37, 0.52	19.95	0.61	0.56, 0.67	29.35
V_T_ (L) at VAT	0.62	0.58, 0.65	39.73	0.48	0.39, 0.57	35.88
*f*_R_ (breaths/min)	8.97	8.42, 9.53	26.90	14.02	13.57, 14.47	39.37
*f*_R_ (breaths/min) at VAT	5.91	5.74, 6.08	28.99	6.12	4.28, 7.95	24.69
HR (beats/min)	16.54	13.57, 19.51	11.25	29.57^a^	24.73, 34.41	21.84^a^
HR (beats/min) at VAT	11.46	9.94, 12.97	11.05	14.81	14.02, 15.60	14.60
Work Rate (watts)	23.30	22.07, 24.53	15.77	18.52	9.02, 28.02	12.89
Work Rate (watts) at VAT	23.66	21.10, 26.22	36.89	20.99	18.77, 23.22	30.13
RPE	3.09	2.81, 3.37	19.91	3.18	2.02, 4.34	21.56

Note

SRD = 1.96 × √*SEM* × √2.

*SEM* = √Mean Square Error from Repeated Measures Analysis of Variance.

95% confidence interval = SRD±mean difference between test 1 and test 2.

SRD% = (SRD/Average of test 1 and test 2) × 100.

Abbreviations: CI, Confidence interval; *f*_R_, respiratory frequency; HR, heart rate; RPE, rating of perceived exertion; *SEM*, standard error of the measure; SRD, smallest real difference; VAT, ventilatory anaerobic threshold; V̇CO_2_, carbon dioxide production; V̇O_2,_ oxygen consumption; V_T,_ tidal volume.

^a^ Data missing for one participant.

## 4 DISCUSSION

We evaluated the 2‐day CPET protocol in veterans with GWI and otherwise healthy controls, with an emphasis on characterizing test–retest reliability, systematic bias, and thresholds for clinically meaningful changes in seven CPET parameters directly measured at VAT and peak exercise. Unlike data from previous 2‐day CPET studies in ME/CFS and in other clinical populations endorsing fatigue as a primary symptom (Bouquet et al., 2019; Braam et al., 2013; Campen et al., 2020; Hodges et al., 2018; Keller et al., 2014; Lien et al., 2019; Nelson et al., 2019; Snell et al., 2013; Vanness et al., 2007; Vermeulen et al., 2010), we did not observe differential changes in physiological function as indicated by the lack of group‐by‐time interactions in our RM‐ANOVA models. However, we were able to substantiate the test–retest reliability of the 2‐day CPET by demonstrating that the ICC values ranged from moderate to excellent for both groups (Table 6), apart from WR at VAT which displayed poor test–retest reliability in veterans with GWI. In addition, we did not detect any clear systematic biases across variables with few outliers (Figures 5, 6). Finally, we used the SRD to estimate minimum values necessary to constitute clinically meaningful changes for indicating decrements in physiological function.

### 4.1 Most 2‐day CPET parameters were reliable in GWI, but generalizability to ME/CFS remains to be seen

Studies of physiological and perceptual responses to exercise are susceptible to myriad sources of variability both nonspecific (e.g., demographics, aerobic fitness, prescription medications, and diurnal variation) and specific (e.g., multiple case‐definitions, illness duration and severity, and symptom profile) to people with GWI and ME/CFS. Presumably, some of these factors influence exercise performance, signifying a need for better characterization of test–retest reliability of CPET parameters in these patient groups. After limiting our patient sample to those providing a valid peak effort, only WR at VAT demonstrated an ICC below 0.5, suggesting that 2‐day CPET produced adequate test–retest reliability for most parameters in veterans with GWI. Given the degree of overlapping symptom profiles between GWI and ME/CFS, data on 2‐day CPET reliability would be valuable for contextualizing our findings and for improving comparability among ME/CFS studies. However, in nine prior studies in ME/CFS (Bouquet et al., 2019; Campen et al., 2020; Hodges et al., 2018; Keller et al., 2014; Lien et al., 2019; Nelson et al., 2019; Snell et al., 2013; Vanness et al., 2007; Vermeulen et al., 2010), CPET test–retest reliability was assumed but not measured.

Provided patients meet criteria for valid effort on their first CPET, the 2‐day protocol requires meeting these same criteria during follow‐up testing 24 hr later. Importantly for people with ME/CFS, the timing of the second CPET coincides with debilitating exacerbation of pain and fatigue (Light et al., 2012; White, Light, Hughen, Vanhaitsma, & Light, 2012), potentially further impairing the ability to provide sufficient peak effort. Conversely, our recent work with GWI patients found that the frequency of veterans experiencing symptom exacerbation and the magnitude of the change 24 hr after 30 min of steady‐state cycling at 70% heart rate reserve was considerably lower than what has been reported in ME/CFS (Lindheimer et al., 2020). The high rate of submaximal performance during a maximal test among individuals with ME/CFS (De Becker, Roeykens, Reynders, McGregor, & De Meirleir, 2000), paucity of data on CPET test–retest reliability in this population, as well as our observation of a less frequent and severe PEM response in GWI (Lindheimer et al., 2020) raises doubt about whether test–retest reliability observed here generalizes to ME/CFS. For these reasons, a separate study characterizing the test–retest reliability of CPET may be warranted in ME/CFS before it can be confidently assumed that the 2‐day CPET protocol affords an objective measure of PEM in this population (Stevens et al., 2018).

### 4.2 The SRD is useful for evaluating clinically meaningful changes in individual patients and facilitates between‐study comparisons

A recent review suggests that the 2‐day CPET quantifies changes in physiological function as a measure of PEM and the magnitude of impairment associated with a patient's compromised recovery (Stevens et al., 2018). When using the 2‐day CPET in this capacity, traditional statistical tests, such as the RM‐ANOVA, are useful because they indicate whether changes in the patient group were significantly different from a relatively healthy response (i.e., group‐by‐time interactions). However, findings from 2‐day CPET studies should also be interpreted in the context of established reference values for absolute or percent changes, which provide perspective on whether changes in the patient group are *clinically* meaningful. Given the heterogeneous nature of GWI and ME/CFS, the SRD statistic is well suited to addressing the need for reference values because it is adjusted for the standard error of the measure, thus allowing researchers and clinicians to better distinguish changes in CPET parameters signifying real physiological alterations from false positives arising from measurement error.

Estimating the SRD across seven different CPET parameters (e.g., V̇O_2_, V̇CO_2,_ V_T_, f_R_, HR, WR, and RPE) at VAT and peak, we observed values ranging from 8.76% to 38.6% for GWI veterans (Table 10). These values can be used by providers as a tool to establish clinical relevance in changes in CPET variables for individual cases. For instance, consider the data from a 49‐year‐old male veteran with GWI who participated in this study. On his first CPET, the veteran met criteria for a valid peak effort (RER = 1.16 and HR of 87.5% predicted max) and achieved a peak V̇O_2_ of 2,151.08 ml·min^−1^. Twenty‐four hours later, he performed a second CPET, again meeting effort criteria (RER = 1.18 and HR of 88.7% predicted max) but achieved a peak V̇O_2_ of 1775.82 ml·min^−1^, that is, an absolute reduction of 375.26 ml·min^−1^ and a relative reduction of −17.4%. Using data from Table 10, the SRD for peak V̇O_2_ from Day 1 to Day 2 would need to exceed 454.53 ml·min^−1^ or 21.9%. Therefore, this veteran did not demonstrate a clinically meaningful reduction in peak V̇O_2_, which might also be interpreted as no evidence of PEM.

The SRD can also facilitate between study comparisons and provide perspective about the replicability of 2‐day CPET studies. For instance, separate studies by Snell et al. (2013), Nelson et al. (2019), and van Campen et al. (2020) have observed significant decreases in WR at VAT across test 1 and test 2 in ME/CFS patients. This decrement in WR at VAT, and V̇O_2_ at VAT in other studies (Keller et al., 2014), has been interpreted as a lowering of the threshold at which anaerobic metabolism accelerates in ME/CFS and failure of aerobic energy‐producing processes in response to exercise stress (Keller et al., 2014). Examining WR at VAT in veterans with GWI, we observed a SRD% of 34.77, meaning that WR at VAT would need to be −34.77% lower on test 2 in comparison to test 1 in order to be clinically meaningful. Prior studies in ME/CFS by Snell et al. (2013), Nelson et al. (2019), and van Campen et al. (2020) observed *statistically* significant reductions in WR at VAT on test 2 in comparison to test 1 of −55.16%, −17.43%, and −30%, respectively. Thus, considering the SRD observed in the present study for veterans with GWI, only Snell et al. (2013) would have observed a *clinically* meaningful reduction in WR at VAT. This raises some doubt about replicability of clinically meaningful changes in WR at VAT across studies, especially considering that this parameter in particular showed the poorest test–retest reliability in our sample of veterans with GWI (ICC = 0.37, 95% CI: −0.17, 0.74).

### 4.3 Limitations and future directions

The primary limitation of this study is our small sample size. However, our sample size is similar to, and in some cases larger than, previous 2‐day CPET studies in clinical populations (Bouquet et al., 2019; Braam et al., 2013; Hodges et al., 2018; Keller et al., 2014; Lien et al., 2019; Nelson et al., 2019; Vanness et al., 2007; Vermeulen et al., 2010). Nevertheless, to obtain practically useful SRD values, it is recommended that a minimum sample size of 15–20 participants is needed, with more recent recommendations advocating for 30–50 participants (Lexell & Downham, 2005). Present study included, we are aware of only one 2‐day CPET study meeting this sample size recommendation (Snell et al., 2013). Thus, although this SRD approach offers promise for establishing reference values that are robust to measurement error, the findings reported here should be viewed as an illustrative example of how the SRD could be applied to 2‐day CPET research in ME/CFS and GWI rather than an official report of reference values that can be used to guide the interpretation of prior and future research. Investigators seeking to use this approach to determine clinically meaningful changes in 2‐day CPET studies will need to establish these SRD values in larger samples than what has been reported here.

Because differences between CPETs 1 and 2 may be attributed to systematic changes caused by the illness itself rather than random error, future attempts to develop SRD reference values for the 2‐day CPET protocol should also consider how best to distinguish typical measurement error in CPET from additive effects of PEM. The SRD is derived from the *SEM*, which is inversely related to reliability (e.g., lower reliability results in higher *SEM* and SRD values), and PEM is associated with increased variability in a given outcome measure, potentially resulting in lower reliability. In the present study, veterans with GWI did not exhibit changes from tests 1 and 2 that significantly differed from healthy controls (i.e., no group‐by‐time interaction), indicating nonsignificant effects of PEM on CPET parameters and by extension that potential additive effects of PEM on measurement error were minimal. This highlights the need for future work testing whether SRD values from repeated CPETs are indeed higher when patients are experiencing PEM compared to when they are not. In people experiencing more severe cases of PEM than observed here (e.g., ME/CFS patients), this idea could be empirically tested by comparing SRD values derived from CPETs separated by 24 hr to those derived from CPETs separated by a time interval long enough for PEM recovery but short enough to minimize other sources of variability in CPET measurement, such as deconditioning or training effects (e.g., CPETs separated by 2 weeks). Additionally, there is a basic need to understand how changes in CPET parameters relate to other measures of PEM. Though it is assumed that greater decrements in physiological function are associated with worsening of symptoms, this has not been investigated in prior studies nor the present investigation.

## 5 CONCLUSION

CPET is a valuable tool for characterizing cardiorespiratory function in healthy and clinical populations. However, studies involving GWI or ME/CFS that use the 2‐day CPET protocol to make inferences about PEM‐related decrements in physiological function should take test–retest reliability and the standard error of the measure into account to avoid potential false positives when interpreting findings. To that end, a better characterization of test–retest reliability and clinically meaningful changes for these patient groups is needed.

## CONFLICT OF INTEREST

The authors declare no conflict of interest.

## AUTHOR CONTRIBUTIONS

Conceptualization: Helene Z. Hill, Gudrun Lange, and Michael J. Falvo. Data curation: Jacquelyn C. Klein‐Adams, and Michael J. Falvo. Formal analysis: Jacob B. Lindheimer, Thomas Alexander, Wei Qian, Benjamin H. Natelson, and Michael J. Falvo. Funding acquisition: Helene Z. Hill, Gudrun Lange, and Michael J. Falvo. Investigation: Helene Z. Hill, Gudrun Lange, and Michael J. Falvo. Methodology: Michael J. Falvo. Project administration: Jacquelyn C. Klein‐Adams and Michael J. Falvo. Supervision: Michael J. Falvo. Writing – original draft: Jacob B. Lindheimer, Thomas Alexander, Wei Qian, Jacquelyn C. Klein‐Adams, Gudrun Lange, Benjamin H. Natelson, Dane B. Cook, Helene Z. Hill, and Michael J. Falvo. Writing – review & editing: Jacob B. Lindheimer, Thomas Alexander, Wei Qian, Jacquelyn C. Klein‐Adams, Gudrun Lange, Benjamin H. Natelson, Dane B. Cook, Helene Z. Hill, and Michael J. Falvo.

## References

[phy214564-bib-0100] American College of Sports Medicine , 2013 ACSM's guidelines for exercise testing and prescription. Philadelphia, PA: Lippincott Williams & Wilkins. 10.1249/JSR.0b013e31829a68cf 23851406

[phy214564-bib-0001] Balady, G. J. , Arena, R. , Sietsema, K. , Myers, J. , Coke, L. , Fletcher, G. F. , … Milani, R. V. (2010). Clinician’s guide to cardiopulmonary exercise testing in adults: A scientific statement from the American Heart Association. Circulation, 122(2), 191–225. 10.1161/CIR.0b013e3181e52e69 20585013

[phy214564-bib-0002] Beaver, W. L. , Wasserman, K. , & Whipp, B. J. (1986). A new method for detecting anaerobic threshold by gas exchange. Journal of Applied Physiology Bethesda MD 1985, 60(6), 2020–2027. 10.1152/jappl.1986.60.6.2020 3087938

[phy214564-bib-0003] Bland, J. M. , & Altman, D. G. (1999). Measuring agreement in method comparison studies. Statistical Methods in Medical Research, 8, 135–160. 10.1177/096228029900800204 10501650

[phy214564-bib-0004] Bouquet, J. , Li, T. , Gardy, J. L. , Kang, X. , Stevens, S. , Stevens, J. , … Chiu, C. Y. (2019). Whole blood human transcriptome and virome analysis of ME/CFS patients experiencing post‐exertional malaise following cardiopulmonary exercise testing. PLoS One, 14(3), e0212193 10.1371/journal.pone.0212193 30897114 PMC6428308

[phy214564-bib-0005] Braam, A. W. E. , de Haan, S. N. , Vorselaars, A. , Rijkers, G. T. , Grutters, J. C. , van den Elshout, F. , & Korenromp, I. (2013). Influence of repeated maximal exercise testing on biomarkers and fatigue in sarcoidosis. Brain, Behavior, and Immunity, 33, 57–64. 10.1016/j.bbi.2013.05.006 23727274

[phy214564-bib-0006] Broderick, G. , Ben‐Hamo, R. , Vashishtha, S. , Efroni, S. , Nathanson, L. , Barnes, Z. , … Klimas, N. (2013). Altered immune pathway activity under exercise challenge in Gulf War Illness: An exploratory analysis. Brain, Behavior, and Immunity, 28, 159–169. 10.1016/j.bbi.2012.11.007 23201588

[phy214564-bib-0007] Broderick, G. , Kreitz, A. , Fuite, J. , Fletcher, M. A. , Vernon, S. D. , & Klimas, N. (2011). A pilot study of immune network remodeling under challenge in Gulf War Illness. Brain, Behavior, and Immunity, 25(2), 302–313. 10.1016/j.bbi.2010.10.011 20955779

[phy214564-bib-0008] Chen, Y. , Meyer, J. N. , Hill, H. Z. , Lange, G. , Condon, M. R. , Klein, J. C. , … Falvo, M. J. (2017). Role of mitochondrial DNA damage and dysfunction in veterans with Gulf War Illness. PLoS One, 12(9), e0184832. 28910366 10.1371/journal.pone.0184832 PMC5599026

[phy214564-bib-0009] Clayton, E. W. (2015). Beyond myalgic encephalomyelitis/chronic fatigue syndrome: An IOM report on redefining an illness. JAMA, 313(11), 1101–1102. 10.1001/jama.2015.1346 25668027

[phy214564-bib-0010] Cohen, J. (1988). Statistical power analysis for the behavioral sciences, 2nd ed Hillsdale, NJ: Lawrence Erlbaum Associates.

[phy214564-bib-0011] Cook, D. B. , Nagelkirk, P. R. , Peckerman, A. , Poluri, A. , Lamanca, J. J. , & Natelson, B. H. (2003). Perceived exertion in fatiguing illness: Gulf war veterans with chronic fatigue syndrome. Medicine & Science in Sports & Exercise, 35(4), 569–574. 10.1249/01.MSS.0000058438.25278.33 12673138

[phy214564-bib-0012] Cook, D. B. , Stegner, A. J. , & Ellingson, L. D. (2010). Exercise alters pain sensitivity in Gulf War veterans with chronic musculoskeletal pain. Journal of Pain, 11(8), 764–772. 20338824 10.1016/j.jpain.2009.11.010

[phy214564-bib-0013] Craig, C. L. , Marshall, A. L. , Sjostrom, M. , Bauman, A. E. , Booth, M. L. , Ainsworth, B. E. , … Oja, P. (2003). International physical activity questionnaire: 12‐country reliability and validity. Medicine and Science in Sports and Exercise, 35(8), 1381–1395. 10.1249/01.MSS.0000078924.61453.FB 12900694

[phy214564-bib-0014] De Becker, P. , Roeykens, J. , Reynders, M. , McGregor, N. , & De Meirleir, K. (2000). Exercise capacity in chronic fatigue syndrome. Archives of Internal Medicine, 160(21), 3270–3277. 10.1001/archinte.160.21.3270 11088089

[phy214564-bib-0015] Dvir, Z. (2015). Difference, significant difference and clinically meaningful difference: The meaning of change in rehabilitation. Journal of Exercise Rehabilitation, 11(2), 67–73. 10.12965/jer.150199 25960978 PMC4415752

[phy214564-bib-0017] Fletcher, G. F. , Ades, P. A. , Kligfield, P. , Arena, R. , Balady, G. J. , Bittner, V. A. , … Williams, M. A. (2013). Exercise standards for testing and training: A scientific statement from the American Heart Association. Circulation, 128(8), 873–934. 10.1161/cir.0b013e31829b5b44 23877260

[phy214564-bib-0018] Fritz, C. O. , Morris, P. E. , & Richler, J. J. (2012). Effect size estimates: Current use, calculations, and interpretation. Journal of Experimental Psychology: General, 141(1), 2–18. 10.1037/a0024338 21823805

[phy214564-bib-0019] Fukuda, K. , Straus, S. E. , Hickie, I. , Sharpe, M. C. , Dobbins, J. G. , & Komaroff, A. (1994). The chronic fatigue syndrome: A comprehensive approach to its definition and study. Annals of Internal Medicine, 121(12), 953–959. 7978722 10.7326/0003-4819-121-12-199412150-00009

[phy214564-bib-0020] Heine, M. , van den Akker, L. E. , Verschuren, O. , Visser‐Meily, A. , & Kwakkel, G. (2015). TREFAMS‐ACE Study Group. Reliability and responsiveness of cardiopulmonary exercise testing in fatigued persons with multiple sclerosis and low to mild disability. PLoS One, 10(3), e0122260 10.1371/journal.pone.0122260 25789625 PMC4366200

[phy214564-bib-0021] Hodges, L. D. , Nielsen, T. , & Baken, D. (2018). Physiological measures in participants with chronic fatigue syndrome, multiple sclerosis and healthy controls following repeated exercise: A pilot study. Clinical Physiology and Functional Imaging, 38(4), 639–644. 10.1111/cpf.12460 28782878

[phy214564-bib-0022] Kazis, L. E. The Veterans SF‐36 Health Status Questionnaire: Development and Application. Veterans Health Administration. 2000;5(1):18.

[phy214564-bib-0023] Kazis, L. , Skinner, K. , Ren, X. , & Perlin, J. Health status and outcomes of veterans: Physical and mental component summary scores. Washington, DC: Department of Veterans Affairs, Veterans Health Administration, Office of Quality and Performance; 1999.

[phy214564-bib-0024] Keller, B. A. , Pryor, J. L. , & Giloteaux, L. (2014). Inability of myalgic encephalomyelitis/chronic fatigue syndrome patients to reproduce VO(2)peak indicates functional impairment. Journal of Translational Medicine, 12, 104 10.1186/1479-5876-12-104 24755065 PMC4004422

[phy214564-bib-0025] Koo, T. K. , & Li, M. Y. (2016). A guideline of selecting and reporting intraclass correlation coefficients for reliability research. Journal of Chiropractic Medicine, 15(2), 155–163. 10.1016/j.jcm.2016.02.012 27330520 PMC4913118

[phy214564-bib-0026] Krupp, L. B. , LaRocca, N. G. , Muir‐Nash, J. , & Steinberg, A. D. (1989). The fatigue severity scale. Application to patients with multiple sclerosis and systemic lupus erythematosus. Archives of Neurology, 46, 1121–1123. 10.1001/archneur.1989.00520460115022 2803071

[phy214564-bib-0027] Lexell, J. E. , & Downham, D. Y. (2005). How to assess the reliability of measurements in rehabilitation. American Journal of Physical Medicine & Rehabilitation, 84(9), 719–723. 10.1097/01.phm.0000176452.17771.20 16141752

[phy214564-bib-0028] Lien, K. , Johansen, B. , Veierød, M. B. , Haslestad, A. S. , Bøhn, S. K. , Melsom, M. N. , … Iversen, P. O. (2019). Abnormal blood lactate accumulation during repeated exercise testing in myalgic encephalomyelitis/chronic fatigue syndrome. Physiological Reports, 7(11), e14138 10.14814/phy2.14138 31161646 PMC6546966

[phy214564-bib-0029] Light, A. R. , Bateman, L. , Jo, D. , Hughen, R. W. , VanHaitsma, T. A. , White, A. T. , & Light, K. C. (2012). Gene expression alterations at baseline and following moderate exercise in patients with chronic fatigue syndrome and fibromyalgia syndrome. Journal of Internal Medicine, 271(1), 64–81. 10.1111/j.1365-2796.2011.02405.x 21615807 PMC3175315

[phy214564-bib-0030] Lindheimer, J. B. , Cook, D. B. , Klein‐Adams, J. C. , Qian, W. , Hill, H. Z. , Lange, G. , … Falvo, M. J. (2019). Veterans with Gulf War Illness exhibit distinct respiratory patterns during maximal cardiopulmonary exercise. PLoS One, 14(11), e0224833 10.1371/journal.pone.0224833 31714907 PMC6850551

[phy214564-bib-0031] Lindheimer, J. B. , Stegner, A. J. , Wylie, G. R. , Klein‐Adams, J. C. , Almassi, N. E. , Ninneman, J. V. , … Cook, D. B. (2020). Post‐exertional malaise in veterans with Gulf War Illness. International Journal of Psychophysiology, 147, 202–212. 10.1016/j.ijpsycho.2019.11.008 31786249 PMC6957714

[phy214564-bib-0032] Nelson, M. J. , Buckley, J. D. , Thomson, R. L. , Clark, D. , Kwiatek, R. , & Davison, K. (2019). Diagnostic sensitivity of 2‐day cardiopulmonary exercise testing in Myalgic Encephalomyelitis/Chronic Fatigue Syndrome. Journal of Translational Medicine, 17(1), 80 10.1186/s12967-019-1836-0 30871578 PMC6417168

[phy214564-bib-0033] Rayhan, R. U. , Raksit, M. P. , Timbol, C. R. , Adewuyi, O. , Vanmeter, J. W. , & Baraniuk, J. N. (2013). Prefrontal lactate predicts exercise‐induced cognitive dysfunction in Gulf War Illness. American Journal of Translational Research, 5(2), 212–223. 23573365 PMC3612516

[phy214564-bib-0034] Rayhan, R. U. , Stevens, B. W. , Raksit, M. P. , Ripple, J. A. , Timbol, C. R. , Adewuyi, O. , … Baraniuk, J. N. (2013). Exercise Challenge in Gulf War Illness Reveals Two Subgroups with Altered Brain Structure and Function. PLoS One, 8(6), e63903. 23798990 10.1371/journal.pone.0063903 PMC3683000

[phy214564-bib-0035] Robergs, R. A. , Dwyer, D. , & Astorino, T. (2010). Recommendations for improved data processing from expired gas analysis indirect calorimetry. Sports Medicine, 40(2), 95–111. 10.2165/11319670-000000000-00000 20092364

[phy214564-bib-0036] Smylie, A. , Broderick, G. , Fernandes, H. , Razdan, S. , Barnes, Z. , Collado, F. , … Klimas, N. (2013) . A comparison of sex‐specific immune signatures in Gulf War Illness and Chronic Fatigue Syndrome. BMC Immunology, 14(1), 1–14. 10.1186/1471-2172-14-29 23800166 PMC3698072

[phy214564-bib-0037] Snell, C. R. , Stevens, S. R. , Davenport, T. E. , & Van Ness, J. M. (2013). Discriminative validity of metabolic and workload measurements for identifying people with chronic fatigue syndrome. Physical Therapy, 93(11), 1484–1492. 10.2522/ptj.20110368 23813081

[phy214564-bib-0038] Steele, L. (2000). Prevalence and patterns of Gulf War illness in Kansas veterans: Association of symptoms with characteristics of person, place, and time of military service. American Journal of Epidemiology, 152(10), 992–1002. 11092441 10.1093/aje/152.10.992

[phy214564-bib-0039] Stevens, S. , Snell, C. , Stevens, J. , Keller, B. , & VanNess, J. M. (2018). Cardiopulmonary exercise test methodology for assessing exertion intolerance in myalgic encephalomyelitis/chronic fatigue syndrome. Frontiers in Pediatrics, 6, 242 10.3389/fped.2018.00242 30234078 PMC6131594

[phy214564-bib-0040] Taylor, H. L. , Buskirk, E. , & Henschel, A. (1955). Maximal oxygen intake as an objective measure of cardio‐respiratory performance. Journal of Applied Physiology, 8(1), 73–80. 13242493 10.1152/jappl.1955.8.1.73

[phy214564-bib-0041] van Campen, C. , Rowe, P. C. , & Visser, F. C. (2020). Validity of 2‐day cardiopulmonary exercise testing in male patients with myalgic encephalomyelitis/chronic fatigue syndrome. Advances in Physical Education, 10(01), 68–80. 10.4236/ape.2020.101007

[phy214564-bib-0042] Vanness, J. M. , Snell, C. R. , & Stevens, S. R. (2007). Diminished cardiopulmonary capacity during post‐exertional malaise. Journal of Chronic Fatigue Syndrome, 14(2), 77–85. 10.1300/J092v14n02_07

[phy214564-bib-0043] Vermeulen, R. C. , Kurk, R. M. , Visser, F. C. , Sluiter, W. , & Scholte, H. R. (2010). Patients with chronic fatigue syndrome performed worse than controls in a controlled repeated exercise study despite a normal oxidative phosphorylation capacity. Journal of Translational Medicine, 8, 93 10.1186/1479-5876-8-93 20937116 PMC2964609

[phy214564-bib-0044] Ware, J. E. , Kosinski, M. , Bjorner, J. B. , Turner‐Bowker, D. M. , Gandek, B. , & Maruish, M. E. (2007). User’s manual for the SF‐36v2(TM) health survey (2nd ed.). Lincoln, RI: QualityMetric Incorporated; [date unknown].

[phy214564-bib-0045] Whistler, T. , Fletcher, M. A. , Lonergan, W. , Zeng, X.‐R. , Lin, J.‐M. , LaPerriere, A. , … Klimas, N. G. (2009). Impaired immune function in Gulf War Illness. BMC Medical Genomics, 2, 12. 19265525 10.1186/1755-8794-2-12 PMC2657162

[phy214564-bib-0046] White, A. T. , Light, A. R. , Hughen, R. W. , Vanhaitsma, T. A. , & Light, K. C. (2012). Differences in metabolite‐detecting, adrenergic, and immune gene expression after moderate exercise in patients with chronic fatigue syndrome, patients with multiple sclerosis, and healthy controls. Psychosomatic Medicine, 74(1), 46–54. 10.1097/PSY.0b013e31824152ed 22210239 PMC3256093

[phy214564-bib-0047] White, R. F. , Steele, L. , O'Callaghan, J. P. , Sullivan, K. , Binns, J. H. , Golomb, B. A. , … Grashow, R. (2016).Recent research on Gulf War illness and other health problems in veterans of the 1991 Gulf War: Effects of toxicant exposures during deployment. Cortex, 74, 449–475. 10.1016/j.cortex.2015.08.022 26493934 PMC4724528

